# Supplementary material for: Oncological and functional outcomes of high-risk and very high-risk prostate cancer patients after robot-assisted radical prostatectomy
Source: PLoS One. 2023 Mar 3;18(3):e0282494. doi: 10.1371/journal.pone.0282494 (PMC9983825; doi:10.1371/journal.pone.0282494)
Supplement: S1 File — (DOCX) [file pone.0282494.s001.docx]

**Supplementary Table S1**

Preoperative, perioperative, pathological, and postoperative characteristics of prostate cancer patients (the entire study cohort, n = 100) in four risk groups

|  | | | Total | Risk group | | | | *p*-value | Post hoc |
| --- | --- | --- | --- | --- | --- | --- | --- | --- | --- |
|  |  |  |  | Low* (A) | Intermediate (B) | High (C) | Very high (D) |  |  |
| Numbers | | | 100 (100.00%) | 20 (20.00%) | 33 (33.00%) | 11 (11.00%) | 36 (36.00%) |  |  |
| Preoperative | | | | | | | | | |
|  | Age | | 69.7 ± 7.4 | 68.5 ± 9.0 | 67.5 ± 6.5 | 71.1 ± 5.8 | 71.8 ± 7.3 | 0.081 |  |
|  | TPV (mL) | | 36.3 ± 14.6 | 32.9 ± 11.8 | 33.6 ± 12.1 | 36.3 ± 17.6 | 40.7 ± 16.5 | 0.133 |  |
|  | iPSA, median (IQR) (ng/mL) | | 12.8 (7.3–25.7) | 6.5 (5.2–8.1) | 10.0 (6.3–14.4) | 25.9 (15.3–34.4) | 27.8 (15.4–51.6) | **<0.001** | (A) vs. (B), (A) vs. (C), (A) vs. (D), (B) vs. (C), (B) vs. (D) |
|  | bGleason score | 6 | 58 (58.00%) | 20 (100.00%) | 20 (60.60%) | 7 (63.60%) | 11 (30.60%) |  |  |
|  |  | 7 | 27 (27.00%) | 0 (0.00%) | 13 (39.40%) | 1 (9.10%) | 13 (36.10%) |  |  |
|  |  | ≥8 | 15 (15.00%) | 0 (0.00%) | 0 (0.00%) | 3 (27.30%) | 12 (33.30%) |  |  |
|  | BMI (kg/m2) | | 25.9 ± 3.2 | 24.7 ± 3.2 | 26.6 ± 2.6 | 26.5 ± 3.7 | 25.7 ± 3.4 | 0.153 |  |
|  | MUL (mm) | | 12.5 ± 2.5 | 12.2 ± 3.0 | 12.9 ± 2.2 | 12.3 ± 2.6 | 12.4 ± 2.4 | 0.783 |  |
|  | Preoperative ADT | | 11 (11.00%) | 1 (5.00%) | 1 (3.00%) | 1 (9.10%) | 8 (22.20%) | 0.056 |  |
|  | Preoperative TURP/TUIP | | 24 (24.00%) | 6 (30.00%) | 7 (21.20%) | 2 (18.20%) | 9 (25.00%) | 0.859 |  |
| Perioperative | | | | | | | | | |
|  | Overall operative time (mins) | | 191.6 ± 30.5 | 183.7 ± 31.6 | 193.5 ± 24.7 | 198.8 ± 43.8 | 192.1 ± 30.5 | 0.557 |  |
|  | Console time (mins) | | 140.6 ± 33.6 | 136.8 ± 20.6 | 140.7 ± 37.4 | 146.8 ± 42.8 | 140.6 ± 33.9 | 0.891 |  |
|  | Blood loss (mL) | | 107.6 ± 79.9 | 90.0 ± 52.8 | 122.8 ± 80.1 | 140.9 ± 141.1 | 93.8 ± 63.6 | 0.162 |  |
|  | NVB preservation | | 43 (43.00%) | 17 (85.00%) | 20 (60.60%) | 1 (9.10%) | 5 (13.90%) | **<0.001** |  |
| Pathological results | | | | | | | | | |
|  | pT stage | pT2 | 58 (58.00%) | 16 (80.00%) | 29 (87.90%) | 6 (54.50%) | 7 (19.40%) | **<0.001** |  |
|  |  | pT3 | 40 (40.00%) | 4 (20.00%) | 4 (12.10%) | 5 (45.50%) | 27 (75.00%) |  |  |
|  |  | pT4 | 2 (2.00%) | 0 (0.00%) | 0 (0.00%) | 0 (0.00%) | 2 (5.60%) |  |  |
|  | pGleason score | 6 | 41 (41.00%) | 14 (70.00%) | 14 (42.40%) | 4 (36.40%) | 9 (25.00%) | **0.001** |  |
|  |  | 7 | 41 (41.00%) | 6 (30.00%) | 16 (48.50%) | 7 (63.60%) | 12 (33.30%) |  |  |
|  |  | ≥8 | 18 (18.00%) | 0 (0.00%) | 3 (9.10%) | 0 (0.00%) | 15 (41.70%) |  |  |
|  | Lymph node involvement | | 7 (7.10%) | 0 (0.00%) | 0 (0.00%) | 2 (18.20%) | 5 (14.30%) | **0.01** |  |
|  | Positive surgical margin | | 18 (18.00%) | 2 (10.00%) | 1 (3.00%) | 1 (9.10%) | 14 (38.90%) | **0.001** |  |
|  | Upstaging | | 40 (40.00%) | 14 (70.00%) | 10 (30.30%) | 7 (63.60%) | 9 (25.00%) | **0.001** |  |
|  | Upgrading | | 32 (32.00%) | 6 (30.00%) | 11 (33.30%) | 4 (36.40%) | 11 (30.60%) | 0.403 |  |
| Postoperative | | | | | | | | | |
|  | Adjuvant radiotherapy | | 30 (30.00%) | 2 (10.00%) | 5 (15.20%) | 4 (36.40%) | 19 (52.80%) | **0.001** |  |
|  | Adjuvant ADT | | 21 (21.00%) | 2 (10.00%) | 2 (6.10%) | 2 (18.20%) | 15 (41.70%) | **0.002** |  |
|  | Follow-up duration, median (range) (months) | | 26.4 (3.3–71.3) | 28.4 (6.1–64.2) | 27.5 (3.3–61.7) | 24.5 (11.5–58.2) | 23.5 (4.3–71.3) | 0.924 |  |

ADT, androgen deprivation therapy; bGleason score, biopsy Gleason score; BMI, body mass index; iPSA, initial serum prostate-specific antigen; IQR, interquartile range; MUL, membranous urethra length; NVB, neurovascular bundles; pGleason score, pathological Gleason score; pT staging, pathological T staging; TPV, total prostate volume; TUIP, transurethral incision of the prostate; TURP, transurethral resection of the prostate.

* Including the very low-risk group.

**Supplementary Table S2**

Preoperative, perioperative, pathological, and postoperative characteristics of prostate cancer patients with functional documentation (n = 71)

|  | | | Total | Risk group | | *p*-value |
| --- | --- | --- | --- | --- | --- | --- |
|  |  |  |  | <High Risk | ≥High Risk |  |
| Numbers | | | 71 (100.00%) | 38 (53.50%) | 33 (46.50%) |  |
| Preoperative | | | | | | |
|  | Age | | 69.8 ± 7.7 | 68.2 ± 8.2 | 71.5 ± 6.9 | 0.077 |
|  | TPV (mL) | | 35.0 ± 15.2 | 30.4 ± 8.7 | 40.3 ± 19.0 | **0.005** |
|  | iPSA, median (IQR) (ng/mL) | | 12.3 (7.3 ± 25.9) | 8.1 (5.5–10.3) | 29.2 (17.5 ± 80.3) | **<0.001** |
|  | bGleason score | 6 | 43 (60.60%) | 29 (76.30%) | 14 (42.40%) | **<0.001** |
|  |  | 7 | 17 (23.90%) | 9 (23.70%) | 8 (24.20%) |  |
|  |  | ≥8 | 11 (15.50%) | 0 (0.00%) | 11 (33.30%) |  |
|  | BMI (kg/m2) | | 25.7 ± 3.3 | 26.0 ± 3.2 | 25.2 ± 3.4 | 0.3 |
|  | MUL (mm) | | 12.4 ± 2.4 | 12.5 ± 2.5 | 12.3 ± 2.3 | 0.804 |
|  | Preoperative ADT | | 10 (14.10%) | 2 (5.30%) | 8 (24.20%) | **0.022** |
|  | Preoperative TURP/TUIP | | 16 (22.50%) | 10 (26.30%) | 6 (18.20%) | 0.413 |
| Perioperative | | | | | | |
|  | Overall operative time (mins) | | 190.4 ± 30.2 | 187.2 ± 26.8 | 194.1 ± 33.6 | 0.337 |
|  | Console time (mins) | | 147.3 ± 31.3 | 146.9 ± 30.1 | 147.8 ± 33.2 | 0.909 |
|  | Blood loss (mL) | | 107.6 ± 72.6 | 117.0 ± 74.8 | 97.1 ± 69.8 | 0.255 |
|  | NVB preservation | | 28 (39.40%) | 24 (63.20%) | 4 (12.10%) | **<0.001** |
| Pathological results | | | | | | |
|  | pT stage | pT2 | 41 (57.70%) | 32 (84.20%) | 9 (27.30%) | **<0.001** |
|  |  | pT3 | 30 (42.30%) | 6 (15.80%) | 24 (72.70%) |  |
|  |  | pT4 | 0 (0%) | 0 (0%) | 0 (0%) |  |
|  | pGleason score | 6 | 32 (45.10%) | 21 (55.30%) | 11 (33.30%) | **0.005** |
|  |  | 7 | 28 (39.40%) | 16 (42.10%) | 12 (36.40%) |  |
|  |  | ≥8 | 11 (15.50%) | 1 (2.60%) | 10 (30.30%) |  |
|  | Lymph node involvement | | 5 (7.00%) | 0 (0.00%) | 5 (15.20%) | **0.004** |
|  | Positive surgical margin | | 11 (15.50%) | 1 (2.60%) | 10 (30.30%) | **0.002** |
| Postoperative | | | | | | |
|  | Adjuvant radiotherapy | | 19 (26.80%) | 5 (13.20%) | 14 (42.40%) | **0.005** |
|  | Adjuvant ADT | | 14 (19.70%) | 2 (5.30%) | 12 (36.40%) | **0.002** |
|  | Follow-up duration, median (range) (months) | | 37.6 (4.3–71.3) | 36.8 (12.0–64.2) | 38.2 (4.3–71.3) | 0.912 |

ADT, androgen deprivation therapy; bGleason score, biopsy Gleason score; BMI, body mass index; iPSA, initial serum prostate-specific antigen; IQR, interquartile range; MUL, membranous urethra length; NVB, neurovascular bundles; pGleason score, pathological Gleason score; pT staging, pathological T staging; TPV, total prostate volume; TUIP, transurethral incision of the prostate; TURP, transurethral resection of the prostate.

**Supplementary Table S3**

Univariable and multivariable logistic regression models of risks of urgency urinary incontinence in prostate cancer patients with functional documentation (n = 71) 1 week, 6 months, and 12 months after robot-assisted radical prostatectomy

|  | | | Urgency Urinary Incontinence at 1 Week* | | | | Urgency Urinary Incontinence at 6 Months | | | | Urgency Urinary Incontinence at 12 Months | | | |
| --- | --- | --- | --- | --- | --- | --- | --- | --- | --- | --- | --- | --- | --- | --- |
|  |  |  | Crude | | Adjusted | | Crude | | Adjusted | | Crude | | Adjusted | |
|  |  |  | OR [95% CI] | *p-*value | OR [95% CI] | *p-*value | OR [95% CI] | *p-*value | OR [95% CI] | *p-*value | OR [95% CI] | *p-*value | OR [95% CI] | p-value |
| Preoperative | | | | | | | | | | | | | | |
|  | Age at OP | | 1.02 [0.96–1.08] | 0.591 | – | – | 0.97 [0.87–1.08] | 0.599 | – | – | 1.09 [0.94–1.26] | 0.239 | – | – |
|  | TPV | | 1.00 [0.97–1.03] | 0.919 | – | – | 1.00 [0.95–1.06] | 0.932 | – | – | 1.03 [0.98–1.09] | 0.202 | – | – |
|  | iPSA | | 1.01 [1.00–1.03] | 0.169 | – | – | 0.99 [0.95–1.03] | 0.508 | – | – | 0.99 [0.96–1.03] | 0.719 | – | – |
|  | Biopsy Gleason score | 6 | Ref | Ref | – | – | Ref | Ref | – | – | Ref | Ref | – | – |
|  |  | 7 | 2.05 [0.65–6.50] | 0.222 | – | – | 0.83 [0.08–8.62] | 0.878 | – | – | 2.73 [0.35–21.17] | 0.336 | – | – |
|  |  | ≥8 | 2.77 [0.72–10.72] | 0.140 | – | – | 2.96 [0.43–20.41] | 0.27 | – | – | 0.00 [0.00–10.00] | 0.999 | – | – |
|  | BMI | | 1.00 [0.87–1.16] | 0.970 | – | – | 1.28 [0.98–1.68] | 0.073 | – | – | 1.22 [0.89–1.67] | 0.218 | – | – |
|  | MUL | | 0.78 [0.62–0.99] | **0.038** | – | – | 1.02 [0.67–1.57] | 0.913 | – | – | 0.92 [0.60–1.41] | 0.687 | – | – |
|  | Risk group | <High–Risk | Ref | Ref | – | – | Ref | Ref | – | – | Ref | Ref | – | – |
|  |  | ≥High–Risk | 2.31 [0.87–6.15] | 0.094 | – | – | 1.17 [0.22–6.22] | 0.857 | – | – | 1.16 [0.15–8.74] | 0.885 | – | - |
|  | Preoperative ADT | | 2.86 [0.73–11.26] | 0.133 | – | – | 1.24 [0.13–11.92] | 0.850 | – | – | 0.00 [0.00–10.00] | 0.999 | – | – |
|  | Preoperative TURP/TUIP | | 2.64 [0.85–8.24] | 0.094 | – | – | 4.00 [0.72–22.16] | 0.112 | – | – | 1.16 [0.11–11.94] | 0.903 | – | – |
| Perioperative | | | | | | | | | | | | | | |
|  | Overall operative time | | 1.00 [0.99–1.02] | 0.859 | – | – | 1.02 [0.99–1.05] | 0.199 | – | – | 1.00 [0.97–1.04] | 0.817 | – | – |
|  | Console time | | 1.00 [0.98–1.01] | 0.689 | – | – | 1.02 [0.99–1.05] | 0.151 | – | – | 1.01 [0.98–1.04] | 0.555 | – | – |
|  | Blood loss | | 1.00 [0.99–1.01] | 0.996 | – | – | 1.00 [0.98–1.01] | 0.574 | – | – | 1.01 [1.00–1.02] | 0.177 | – | – |
|  | NVB preservation | | 0.51 [0.18–1.40] | 0.188 | – | – | 0.75 [0.13–4.40] | 0.750 | – | – | 0.00 [0.00–10.00] | 0.998 | – | – |
| Pathological results | | | | | | | | | | | | | | |
|  | pT stage | pT2 | Ref | Ref |  |  | Ref | Ref |  |  | Ref | Ref |  |  |
|  |  | ≥pT3 | 0.91 [0.34–2.39] | 0.840 | – | – | 0.66 [0.11–3.87] | 0.646 | – | – | 0.44 [0.04–4.42] | 0.483 | – | – |
|  | Pathological Gleason score | 6 | Ref | Ref | – | – | Ref | Ref | – | – | Ref | Ref | – | – |
|  |  | 7 | 1.43 [0.50–4.07] | 0.501 | – | – | 0.36 [0.04–3.65] | 0.386 | – | – | 3.72 [0.36–37.99] | 0.268 | – | – |
|  |  | ≥8 | 1.09 [0.26–4.55] | 0.905 | – | – | 2.15 [0.31–14.94] | 0.440 | – | – | 0.00 [0.00–10.00] | 0.999 | – | – |
|  | Lymph node involvement | | 0.39 [0.04–3.64] | 0.404 | – | – | 0.00 [0.00–10.00] | 0.999 | – | – | 5.25 [0.44–62.60] | 0.190 | – | – |
|  | Positive margin | | 3.50 [0.92–13.38] | 0.067 | – | – | 1.10 [0.12–10.44] | 0.934 | – | – | 1.90 [0.18–20.14] | 0.594 | – | – |
| Postoperative | | | | | | | | | | | | | | |
|  | Adjuvant radiotherapy | | NA | NA | NA | NA | 1.41 [0.24–8.42] | 0.705 | – | – | 0.91 [0.09–9.30] | 0.935 | – | – |
|  | Adjuvant ADT | | NA | NA | NA | NA | 0.00 [0.00–10.00] | 0.999 | – | – | 1.39 [0.13–14.41] | 0.785 | – | – |

ADT, androgen deprivation therapy; bGleason score, biopsy Gleason score; BMI, body mass index; <high-risk, below high-risk group; ≥high-risk, high-risk/very high-risk group; iPSA, initial serum prostate-specific antigen; MUL, membranous urethra length; NA, not available; NVB, neurovascular bundles; OR, odds ratio; pGleason score, pathological Gleason score; pT staging, pathological T staging; RaRP, robot-assisted radical prostatectomy; TPV, total prostate volume; TURP, transurethral resection of the prostate; TUIP, transurethral incision of the prostate; UUI, urgency urinary incontinence.

*One week after RaRP, immediately after removal of the urethral catheter.
