# Supplementary material for: Oncological and functional outcomes of high-risk and very high-risk prostate cancer patients after robot-assisted radical prostatectomy
Source: PLoS One. 2023 Mar 3;18(3):e0282494. doi: 10.1371/journal.pone.0282494 (PMC9983825; doi:10.1371/journal.pone.0282494)
Supplement: Attachment — Submitted filename: Response to Reviewers_20230121.docx [file pone.0282494.s002.docx]

**Dear Editor:**

Thank you to give us a chance to revise. We have revised the manuscript based on the comments of the reviewers. Please refer yourself to the revised manuscript WITHOUT track changes version.

**Reviewer #1:**

I would like to thank the editor for this opportunity and the authors foe their efforts. Investigating the outcomes of robot-assisted radical prostatectomy outcomes based on a risk stratification method on included sample of patients and also including the impact of neoadjuvant therapy besides the surgery in this study is interesting and adds to the literature. Although the study notion is not novel, the methodology made the material interesting and highlighting the worse outcomes in high-risk patients leverages the importance of material from a clinical aspect of evaluation. The manuscript needs some revisions prior to finalization and reaching a decision. My comments and suggestions are provided below.

1. The first major concern about this manuscript is the used terms of “high-risk” and “very high-risk” patients with prostate cancer in need of surgery. The used nomenclature is not so usual in the literature in field. It is more questionable when the authors presented the results stratified by “below high-risk” and “high-risk and very high-risk”. Why not reporting the results for “low-risk” and “high-risk” patients as it is common in literature? A revision on this issue might be helpful.

Response: Thank you for the comment.

1. There are at least four risk groups according to NCCN criteria, which was derived from D’Amico risk stratification. We have added the description in the text. (Line 88 – 96)
2. First, the patient numbers in “four groups (low-, intermediate-, high-, very high-risk group)” are small, and might limit the significance for comparison. Second, categorizing intermediate-risk patients into the low-risk group would be misleading given the fact that intermediate risk is still different than low-risk patients in terms of oncological outcomes. Finally, we focused on the oncological and functional outcomes in high-/ very high -risk groups, which were not clearly evaluated before. Therefore, to keep the current stratification with “below high-risk group” and “high-/very high-risk group” is more feasible in this study.

2. Abstract: this section needs more information of the study methods and the definitions of study groups and outcomes in the materials and methods part, to make the following presented results clearer and easy to understand.

Response: Thank you for the comment. We have revised the abstract accordingly to make it clearer and easy to understand. We also added NCCN initial risk stratification and the primary endpoint (continence within year one), and the secondary end-point (BCR-free survival) in the abstract. (Line 25 – 28)

3. Introduction: the part on the review of previous literature and highlighting the gap in studies that is tried to be covered by this study is insufficient and needs more expansion and citation to similar previous studies.

Response: Thank you for the comment. We have revised this section accordingly, and added more references (Ou YC, Yang CK, Wang J, Hung SW, Cheng CL, Tewari AK, et al. The trifecta outcome in 300 consecutive cases of robotic-assisted laparoscopic radical prostatectomy according to D’Amico risk criteria. European Journal of Surgical Oncology. 2013 Jan 1;39(1):107–13.) (Line 71 – 74 & Ref.11)

4. Materials and Methods: The second paragraph introducing the two groups of 5. Statistical analysis: it should be clear whether the authors used “multivariate” or “multivariable” logistic regression models to investigate the predictive factors for postoperative outcomes, since these two are different methods statistically. Also, adding the log-rank test as the statistical test to compare the results of survival analysis is suggested in this part.

Response: Thank you for the comment. We have changed all the “univariate” and “multivariate” to “univariable and multivariable logistic regression”. As for the odds ratio, we have changed it to “crude” and “adjusted” odds instead of “univariate” and “multivariate”. All the p-value in the Kaplan-Meier survival graph was generated with log-rank test. We have reiterated that part in material and methods. (Line 146, 202, 209, 211, 216-217, 238, 307, 319, 322, Table 2, Supplementary Table S3)

6. Results: some results in the tables are stratified by four risk groups and it was not mentioned in the methods what the criteria for this stratification was. One major issue about the results are the survival results presented in figure 1B. Since the provided P-value are between three recruited groups it is not clear whether the results between the high-risk groups with and without AT were significant or not. Adding another plot with only these two groups would be beneficial.

Response: Thank you for the comment.

1. All the patients were stratified according to the NCCN guideline, which was derived from D’Amico risk stratification. We have added the description in the text. (Line 86 – 88)
2. In figure 1B, we have run the Kaplan-Meier survival graph again, only using two subgroups. The log-rank test was still less than 0.05. We have revised figure 1B accordingly (See revised Figure 1).

7. Discussion: although the results are compared with literature well, some studies are missed and it is suggested to included relevant studies about the results of RaRP in high-risk patients to this section (Example: Abdollah F, Sood A, Sammon JD, Hsu L, Beyer B, Moschini M, Gandaglia G, Rogers CG, Haese A, Montorsi F, Graefen M, Briganti A, Menon M. Long-term cancer control outcomes in patients with clinically high-risk prostate cancer treated with robot-assisted radical prostatectomy: results from a multi-institutional study of 1100 patients. Eur Urol. 2015 Sep;68(3):497-505. doi: 10.1016/j.eururo.2015.06.020. Epub 2015 Jun 26. PMID: 26119559.)

Response: Thank you for the comment. We have added the references accordingly. (Line 243 – 245, Ref. 14)

—------------------------------------------------------------------------------------------------------------------

**Reviewer #2:**

Thanks for sending me this analytical work about prostate cancer robotic treatment. First of all congratulations to the authors for their work and achievement, I believe the aims of study are of utmost importance to the designated field but there are some major issues that need to be addressed before proceeding. Would be happy to help with revision and eventually see the paper published.

Majors:

1- The definition of risk (high, low, etc) are not defined while being used throughout the paper

Response: Thank you for the comment. All the patients were stratified according to the NCCN guideline, which was derived from D’Amico risk stratification. We have added the detailed description in the text. (Line 86 – 88)

2- The flow of paper (both scientific and narrative) is hard to follow. Several times I was lost in the middle of results and had to turn back and read from the beginning. In other words, the results section is not organized and is long. They may add some subsections

Response: Thank you for the comment. We have added subsections to make the results more organized and easy to read. (Line 157, 173, 184)

Minors:

1- Authors were too generous with using abbreviations. I strongly suggest using full wording as much as possible

2- I think it is MacOS Big Sur

Response: Thank you for the comment. We have corrected MacOS Big Sur accordingly. As for the excessive abbreviations, we have minimized the use of abbreviations (including stress urinary incontinence [SUI], urgency urinary incontinence [UUI], and biochemical recurrence [BCR])

**Reviewer #3:**

In this paper, BCR-free survival rate as an oncological outcome and stress urinary incontinence (SUI) and Urgency Urinary Incontinence (UUI) as functional outcomes of robot assisted radical prostatectomy (RaRP) were investigated in high-risk and very high-risk prostate cancer patients and were compared to below high-risk group. The BCR-free survival of patients in high-risk and very high-risk group who also received adjuvant therapy was comparable to those in below high-risk group. Also, in long-term follow-up, the rate of SUI was not significantly different between high-risk/ very high-risk and low risk group. Suggesting that RaRP is a safe treatment option in high-risk/ very high-risk prostate cancer patients.

The paper is well-written and the content is interesting to the readers of the journal.

In my opinion, the paper could be accepted after making major/minor revisions as follows:

Major concerns

It is not clear how the patients were assessed in the selected time intervals. Was it through clinic visits?

Response: Thank you for the comment. All the patients were regularly followed up at clinic visits after discharge. The intervals of postoperative clinic visits were monthly within first 3 months followed by every three months, with additional visits if clinically indicated. We have added above description in the text. (Line 118 – 120)

It would be better to mention in the statistical analysis part that data was reported as mean ± SD and median (range).

Response: Thank you for the comment. We have added “The continuous data was reported as mean ± SD (standard deviation) and median (interquartile range).” to statistics analysis part accordingly. (Line 146- 147)

As well as age, the relationship between other characteristics of the 71 patients included for SUI and UUI analysis should be mentioned.

Response: Thank you for the comment. We have added description as follows:

Among the 71 patients, high-risk/very high-risk patients had larger total prostate volume, a higher proportion of receiving preoperative ADT, less percentage of neurovascular bundle preservation, and higher proportion of receiving adjuvant radiotherapy and ADT. (Line 190- 193)

In 5th paragraph of results: odds ratios should come with confidence intervals.

Response: Thank you for the comment. We have added CI to the paragraph. (Line 204- 213)

In tables 1, S1, and S2, the total columns need to be transferred before risk groups as the p-value is related to the comparison of the risk groups.

Response: Thank you for the comment. We have transferred the column in that fashion accordingly. (Table 1, Supplementary Table S1, Supplementary Table S1)

In table 2 and supplementary table 3 heading, the number of patients that were included and analyzed should be mentioned.

Response: Thank you for the comment. We have added (n = 71) in the heading accordingly. (Table 2)

Heading of table 1 and S2 are similar, and their difference is unclear.

Response: Thank you for the comment. We have revised the heading of table S2 to “Preoperative, perioperative, pathological, and postoperative characteristics of prostate cancer patients with functional documentation  (n = 71)”. (Table 1 and Supplementary Table S2)

Figure 2: in the horizontal axis the interval between 0-1 months shouldn’t be equal to 1-3 months or 3-6 months.

Response: Thank you for the comment. We have revised the intervals of the X-axis of figure 2. (Figure 2 revised)

The latest study that authors discussed and referred is for Jul 2021, there are some recent studies for example by Shin et al. on oncological and functional outcomes of RaRP that should be discussed here.

Response: Thank you for the comment. We have cited the article (https://doi.org/10.1186/s12894-022-00998-6) in discussion. (Line 243 – 245, Ref. 14)

Minor concerns

For the reference [(11)] in the material and method part, [] is extra.

Response: Thank you for the comment. We have deleted the “[]” accordingly. (Line 73)

UUI is abbreviation for “Urge/ urgency urinary incontinence” not “urinary urgency incontinence”.

Response: Thank you for the comment. We have corrected all that in to urgency urinary incontinence.

In 4th paragraph of results, after talking about 71 patients included, first the number (%) of patients in each group should be mentioned and then the age and other characteristics.

Response: Thank you for the comment. We have added “Seventy-one patients (71%) in our cohort had documented function outcomes within one year after RaRP.” in the first sentence in the paragraph (Line 185-186). The following sentences were also included in the paragraph as follows:

Of these 71 patients, 38 (53.5%) were in the below high-risk group and 33 (46.5%) were in the high-risk/very high-risk group (Supplementary Table S2). Among the 71 patients, high-risk/very high-risk patients had significantly larger total prostate volume, a significantly higher proportion of preoperative and adjuvant (postoperative) ADT, adjuvant radiotherapy, and a significantly lower percentage of neurovascular bundle preservation. (190 - 193)

Style of reference 4 should be corrected. It’s a published paper and there is no need to mention the website.

Response: Thank you for the comment. We have revised the reference as follows.

Wang Z, Ni Y, Chen J, Sun G, Zhang X, Zhao J, et al. The efficacy and safety of radical prostatectomy and radiotherapy in high-risk prostate cancer: a systematic review and meta-analysis. World J Surg Oncol. 2020 Feb 24;18(1):42. (Ref.4)
